# CD146 is essential for PDGFRβ-induced pericyte recruitment

**DOI:** 10.1007/s13238-017-0484-5

**Published:** 2017-10-16

**Authors:** Jianan Chen, Yongting Luo, Hongxin Huang, Shuilong Wu, Jing Feng, Jingjing Zhang, Xiyun Yan

**Affiliations:** 1 Key Laboratory of Protein and Peptide Pharmaceutical, Institute of Biophysics, Chinese Academy of Sciences, Beijing, 100101 China; 2 College of Life Sciences, University of Chinese Academy of Sciences, Beijing, 100049 China; 3 Beijing Advanced Innovation Center for Food Nutrition and Human Health, China Agricultural University, Beijing, 100193 China; 4 Affiliated Hospital of Guangdong Medical University, Zhanjiang, 524001 China; 5 Laboratory of Developmental Biology and Regenerative Medicine, School of Medicine, South China University of Technology, Guangzhou, 510006 China

**Dear Editor,**

The homeostasis of the central nervous system (CNS) is regulated through the blood-brain barrier (BBB), a protective barrier between the peripheral blood and the brain (Zhao et al., 2015). As vascular mural cells of brain microvessels, pericytes are positioned within the neurovascular unit among endothelial cells (ECs), astrocytes, and neurons. During the embryogenesis, pericytes are recruited to and interacts with the nascent CNS vascular ECs for BBB formation and maturation. The recruitment of pericytes to CNS vessels is hence critical for BBB development. A large amount of evidence has suggested that platelet-derived growth factor B (PDGF-B)/PDGFRβ signaling functions as the major regulatory pathway in governing pericyte recruitment (Bell et al., 2010; Daneman et al., 2010; Sweeney et al., 2016; Chen et al., 2017). Homozygous deletion of either *Pdgfb* or *Pdgfrb* in mouse embryos results in a complete lack of brain pericytes and CNS microhemorrhages, causing embryonic lethality (Daneman et al., 2010). In other studies, partial disruption of PDGF-B/PDGFRβ signaling leads to BBB breakdown and various CNS pathophysiologies associated with pericyte deficiency (Bell et al., 2010; Sweeney et al., 2016). These studies highlight a fundamental role of PDGF-B/PDGFRβ signaling in pericyte recruitment and CNS homeostasis. Limited studies have shown that in fibroblasts or mesenchymal stem cells, the full activation of PDGFRβ requires its association with CD44, TGFβ type I receptor, neuropilin-1, or transglutaminase (Zemskov et al., 2009; Ball et al., 2010; Porsch et al., 2014), indicating that membrane proteins are essential for the functional signaling. However, the regulatory mechanisms governing PDGF-B/PDGFRβ activation and how the activated PDGFRβ induces its intracellular downstream signaling in brain pericytes remain to be elucidated.

CD146, which was originally identified as a novel endothelial biomarker for angiogenesis, has also been found constitutively expressed in pericytes and has subsequently been used for pericyte isolation and characterization (Crisan et al., 2008; Sweeney et al., 2016). Our recent study showed that pericyte-specific deletion of CD146 leads to BBB breakdown and impairment in pericyte recruitment due to the deficiencies of PDGFRβ activation in mice (Chen et al., 2017). However, how pericytic CD146 regulates PDGFRβ activation to transduce its downstream signals properly and efficiently during BBB development is still incompletely unrevealed. In endothelial cells (ECs), CD146 has been reported to activate the Fyn/Fak/paxillin pathway (Anfosso et al., 2001). It could also act as a co-receptor for VEGFR2 to facilitate VEGF-induced VEGFR-2 phosphorylation, AKT/p38 MAPKs/NF-κB activation during angiogenesis, and this relies on its association with the cell cytoskeleton (Jiang et al., 2012). Besides, we have recently identified CD146 as a novel receptor for netrin-1 to promote p38 MAPKs/ERK activation and physiological angiogenesis (Tu et al., 2015). Above studies prompt us to elucidate the mechanisms and roles of CD146 in regulating PDGF-B/PDGFRβ signaling in pericytes during BBB development.

In the present work, we employed various tools, including siRNA to silence the expression of *Cd146*, monoclonal antibody AA98 that specifically inhibits the function of CD146, dimerization-deficient mutant of CD146 or CD146 mutant lacking cytoskeleton binding site, to investigate the precise mechanism of CD146 in controlling PDGF-B/PDGFRβ signaling during BBB development in *in vitro* brain pericytic model and *in vivo* zebrafish model.

To investigate whether CD146 regulates PDGF-B/PDGFRβ signaling, we incubated the pericyte progenitor cell line 10T1/2 with PDGF-B. In parallel, *Cd146* siRNA which specifically knocks down the expression of CD146 was applied as a control. Our results showed that PDGF-B stimulation induced PDGFRβ phosphorylation and activated downstream AKT/JNK/ERK/p38 signaling. This activation was impaired by CD146 knockdown. Furthermore, the inactivation caused by knockdown of CD146 was rescued again by restoring CD146 expression (Figs. 1A and S1A). These results suggest that CD146 plays an important role in the PDGF-B/PDGFRβ pathway.

**Figure 1:**
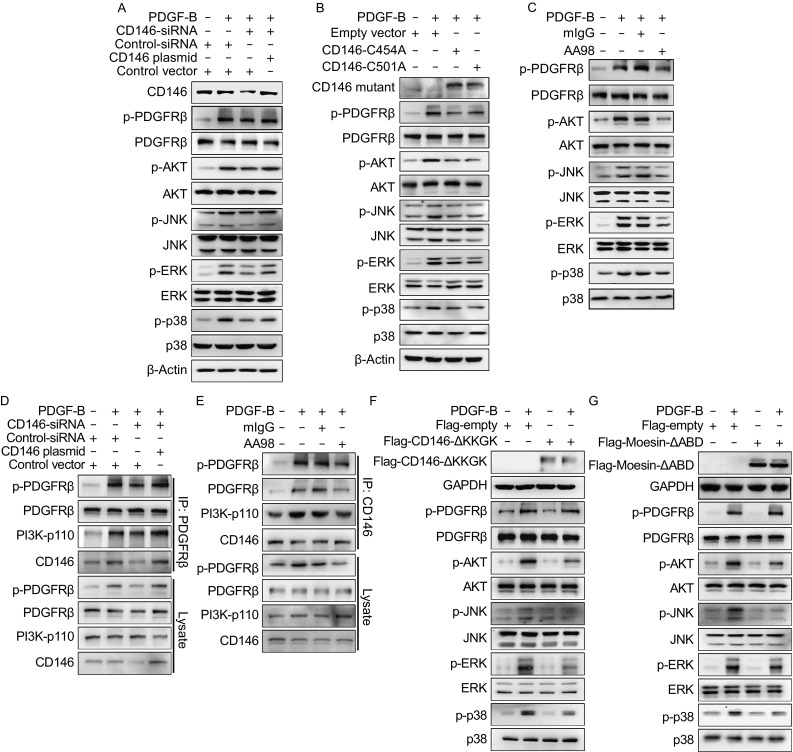
**CD146 is required for PDGFRβ-mediated signal transduction**. (A) 10T1/2 cells were transfected with *Cd146*-siRNA or co-transfected with *Cd146*-siRNA and a *Cd146*-expressing plasmid, and then stimulated with PDGF-B (20 ng/mL). CD146 expression and the activation of PDGFRβ, AKT, JNK, ERK, and p38 were detected by Western blotting. (B) *Cd146*/*C454A* or *Cd146*/*C501A* was transfected into 10T1/2 cells and the activation of PDGFRβ, AKT, JNK, ERK, and p38 induced by PDGF-B (20 ng/mL) was detected by Western blotting. (C) 10T1/2 cells were stimulated with PDGF-B (20 ng/mL) in the presence of mIgG or AA98 (50 μg/mL). The activation of PDGFRβ, AKT, JNK, ERK, and p38 was detected by Western blotting. (D) Co-immunoprecipitation (Co-IP) of endogenous CD146, PDGFRβ, p-PDGFRβ, and p110 induced by PDGF-B (20 ng/mL) was measured after the transfection of 10T1/2 cells with *Cd146*-siRNA or co-transfection with *Cd146*-siRNA and a *Cd146*-expressing plasmid. (E) 10T1/2 cells were stimulated with PDGF-B (20 ng/mL) in the presence of mIgG or AA98 (50 μg/mL). The interactions among endogenous CD146, PDGFRβ, p-PDGFRβ, and p110 were detected by Co-IP. (F) *Cd146-∆KKGK* was transfected transiently into 10T1/2 cells and the activation of PDGFRβ induced by PDGF-B in addition to AKT, JNK, ERK, and p38 signaling was analyzed by western blotting. (G) *Moesin-∆ABD* was transfected transiently into 10T1/2 cells and the activation of PDGFRβ after induction with PDGF-B in addition to AKT, JNK, ERK, and p38 signaling was examined. Data represent three independent experiments

To investigate the impact of CD146 dimerization on the downstream signal transduction of PDGFRβ in pericytes, we transfected 10T1/2 cells with *Cd146*/*C454A* or *Cd146*/*C501A* to block dimerization of mice CD146. Our results showed that the PDGF-B-induced PDGFRβ phosphorylation and the downstream signal transduction were totally inhibited by either *Cd146*/*C454A* or *Cd146*/*C501A* (Figs. 1B and S1B), suggesting that CD146 dimerization is required for PDGF-B/PDGFRβ signaling. To confirm the role of this dimerization on the signal activation of PDGFRβ, we further applied anti-CD146 AA98, which has been reported to abrogate CD146 dimerization (Zheng et al., 2009). The results turned out that PDGF-B-activated PDGFRβ phosphorylation and the AKT/JNK/ERK/p38 signaling were impaired by AA98 treatment, while the mIgG-treated cells were unaffected (Figs. 1C and S1C), indicating that CD146 dimerization plays an essential role in PDGFRβ phosphorylation and AKT/JNK/ERK/p38 signal activation.

Since it has been demonstrated that PDGF-B engagement triggers PDGFRβ phosphorylation and subsequent PI3K recruitment for downstream signal activation, we next investigated whether CD146 was required for PDGFRβ/PI3K complex formation using AA98 and siRNA against *Cd146*. Co-immunoprecipitation revealed that CD146 binds to phosphorylated PDGFRβ (p-PDGFRβ) and PI3K in 10T1/2 cells. This association was enhanced by PDGF-B stimulation, whereas knockdown of *Cd146* (Figs. 1D and S1D) or AA98 pre-treatment (Figs. 1E and S1E) significantly impaired the complex formation. These suggested that CD146 regulates PDGF-B signaling by recruiting p-PDGFRβ and PI3K.

We previously demonstrated that downstream signal transduction and cell migration induced by CD146 requires physical interaction between CD146 and the cytoskeleton, which is mediated by actin-linking ezrin-radixin-moesin (ERM) proteins. The juxtamembrane KKGK motif of CD146 is required for its firm association with ERM proteins (Luo et al., 2012). Therefore, we asked whether this interaction is also essential for PDGFRβ signaling. To this end, a *Cd146* construct lacking the KKGK motif (coding *Cd146-∆KKGK*) or a *Moesin* construct lacking the actin-binding domain (coding *Moesin-∆ABD*) was transfected into 10T1/2 cells. We observed that either *Cd146-∆KKGK* or *Moesin-∆ABD* impaired PDGF-B-induced PDGFRβ downstream signaling without affecting PDGFRβ phosphorylation (Figs. 1F, 1G, S1F, and S1G). In addition, transfection with *Cd146-∆KKGK* or *Moesin-∆ABD* caused a loss of PDGF-B-induced migratory and proliferation ability of 10T1/2 cells (Fig. S2). These suggest that the interaction between CD146 and the cytoskeleton, bridged by ERM proteins, is critical for PDGF-B-induced signal transduction, as well as cell migration and proliferation.

Since it has been reported that the PDGF-B/PDGFRβ signaling is important for pericyte recruitment to the vascular wall, we next used zebrafish (*Danio rerio*) as an *in vivo* model to investigate the impact of CD146 on the pericyte recruitment and the BBB development. For this, *cd146* and/or *pdgfrb* were first knocked down using specific antisense oligonucleotide morpholinos (MOs). Next, fluorescent *in situ* hybridization using *pdgfrb* probe as a pericyte-specific marker was combined to investigate the recruited pericytes. As a result, we found that *cd146*-MO or *pdgfrb*-MO, alone or in combination, resulted in a significant decrease of pericyte recruitment at 72 h post-fertilization (hpf) (Figs. 2A and S3A), demonstrating that CD146 and PDGFRβ synergistically regulate pericyte recruitment.

**Figure 2:**
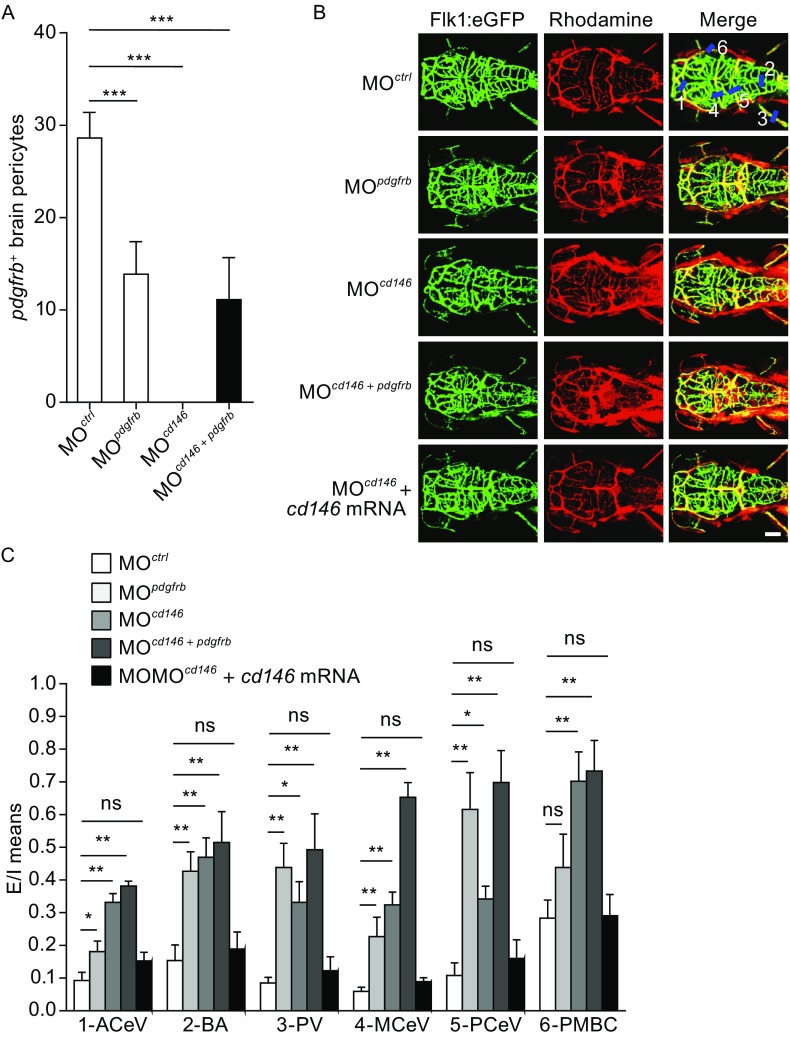
**Knockdown of** ***cd146*** **impairs BBB integrity and pericyte recruitment via PDGF-B signaling in zebrafish**. (A) Quantification of *pdgfrb* ^+^ pericytes attached to cerebrovascular walls in zebrafish injected with *cd146* morpholino, *pdgfrb* morpholino, or co-injected with *cd146* and *pdgfrb* morpholinos. (B) Cerebral microangiography to analyze the BBB permeability of the 72 hpf zebrafish larvae injected with *cd146* morpholino, *pdgfrb* morpholino, or co-injected with *cd146* and *pdgfrb* morpholinos or with *cd146* morpholino and *cd146* mRNA. Scale bar represents 100 μm. (C) Quantification of the Rhodamine signal for the intensity value of the vasculature lumen (I) relative to that out of the cerebral vasculature (E) in different central nervous system vessels of zebrafish injected with morpholinos as indicated. For each analyzed vessel, five positions along the vessel were chosen for calculating the E/I value; *P < 0.05, **P < 0.01, ***P < 0.001. Data represent five independent experiments. ACeV, anterior (rostral) cerebral vein; BA, basilar artery; PV, pectoral vein; MCeV, middle cerebral vein; PCeV, posterior (caudal) cerebral vein; PMBC, primordial midbrain channel

To further investigate the role of CD146 in BBB integrity, we micro-injected the tracing dye of rhodamine B-dextran (70 kDa) into the vasculature of *cd146*-MO zebrafish larvae at 3 dpf, when the function of BBB was initially formed. Our results showed that the dye leaked from the cerebrovasculature into the brain tissues in *cd146*-MO embryos. Knockdown of *pdgfrb* or co-injection of *cd146*-MO and *pdgfrb*-MO resulted in a similar phenotype of leakage. However, rescue of CD146 expression by co-injection of *cd146*-MO with exogenous *cd146* mRNA restored CNS vessel permeability, where the fluorescent dye was retained within the cerebrovasculature 30 min post-injection, similar to that observed in control larvae (Fig. 2B and 2C). This *in vivo* evidence indicates that CD146 is required for the functional formation of the BBB, and functions by regulating PDGFRβ pathway.

While since PDGFRβ is only expressed in pericytes, *pdgfrb* knockdown could not affect cerebral vasculogenesis nor EC number in zebrafish embryos before 56 hpf. In contrast, knockdown of *cd146* or simultaneous treatment with *cd146* and *pdgfrb* MOs resulted in severe defects in the cerebrovasculogenesis (Fig. S3). This is consistent with the early researches showing that CD146 is also expressed in ECs and is required for vascular sprouting and branching in zebrafish (Tu et al., 2015). Since CD146 is expressed in both ECs and pericytes, it is not easy to ascertain a causal relationship between pericytic *cd146* knockdown and BBB integrity using *cd146*-MO zebrafish, as CD146 in ECs is also suppressed. The generation of pericyte-specific *cd146* knockout zebrafish may be further needed to answer this question. However, combining the *in vitro* evidence that siRNA against *Cd146* or anti-CD146 AA98 treatment in pericytes blocked PDGFRβ-mediated signal transduction, we confirmed that CD146 is required for pericyte recruitment by regulating the PDGFRβ pathway.

Our study demonstrated that CD146, a reliable marker of pericytes, participates in PDGF-B/PDGFRβ-induced pericyte recruitment during BBB development. Based on the *in vitro* studies, we conclude that CD146 regulates PDGF-B/PDGFRβ signaling in a multifaceted manner, involving CD146-PDGFRβ binding, CD146 dimerization, PI3K recruitment, and CD146-cytoskeleton binding. Combined with the zebrafish *in vivo* studies showing that either knockdown of *cd146* or *pdgfrb* impaired pericyte recruitment and BBB integrity, we propose a model for CD146 to sequentially regulate PDGF-B/PDGFRβ signaling and the recruitment of pericytes during BBB formation: within the cell membrane, the dimerization of CD146 associates with PDGFRβ to enable efficient PDGF-B-induced receptor auto-phosphorylation, thereby enhancing the recruitment of PI3K-p110 to the p-PDGFRβ; subsequently, the CD146-ERM-cytoskeleton complex functions as scaffold for diverging signaling cascades downstream of PDGFRβ, thus promoting pericyte recruitment and BBB integrity.

Previous studies have proposed several mechanisms through which PDGF-B induces PDGFRβ activation in pericytes, including PDGF ligand diversity and availability as well as interplay with membrane proteins or co-receptors (Andrae et al., 2008). In this study, we observed an association between CD146 and PDGFRβ, which enhances PDGFRβ activation in pericytes. This is consistent with numerous studies demonstrating that the interaction between PDGFRβ and membrane proteins such as neuropilin-1, CD44, TGFβ type I receptor, and transglutaminase, is essential for the functional signaling of PDGFRβ. Therefore, the association with membrane proteins might be a common mechanism exploited by pericytes for PDGFRβ activation. In conclusion, our study identifies　the cell adhesion receptor CD146 as a novel regulator in PDGF-B/PDGFRβ signaling and sheds light on the mechanisms governing pericyte recruitment in both the development of BBB and neurological disorders associated with BBB breakdown.

## Electronic supplementary material

Below is the link to the electronic supplementary material.

Supplementary material 1 (PDF 536 kb)
