# Supplementary material for: CD146 is essential for PDGFRβ-induced pericyte recruitment
Source: Protein Cell. 2017 Oct 16;9(8):743–7. doi: 10.1007/s13238-017-0484-5 (PMC6053352; doi:10.1007/s13238-017-0484-5)
Supplement: Supplementary file 1 — Supplementary material 1 (PDF 536 kb) [file 13238_2017_484_MOESM1_ESM.pdf]

Supplemental materials

Materials and Methods

Antibodies and reagents

The anti-CD146 monoclonal antibody AA98 was described previously (Zhang et al., 2008). Rat anti-mouse CD146 (clone: ME-9F1) was from BioLegend (San Diego, CA, USA). Antibodies against p-p38, p38, p-AKT, AKT, p-ERK, ERK, p-JNK, JNK, p-PDGFRβ, and PDGFRβ were from Cell Signaling Technology (Danvers, MA, USA). An antibody specific for p110 was from Abcam (Cambridge, MA, USA). Recombined PDGF-B was from PeproTech (Rocky Hill, NJ, USA). The control mIgG antibody was purchased from Sigma-Aldrich (St. Louis, MO, USA).

Plasmids, siRNA duplex, and cell transfection

The plasmid (*pcDNA3.1-Cd146*) encoding the full-length sequence of murine CD146 was obtained from Sino Biological Inc. *Cd146* mutant cDNA with respective mutations at cysteines were generated by site-directed mutagenesis using *pcDNA3.1-Cd146* as a template, and were designated CD146/C454A and CD146/C501A. The flag-tagged CD146-ΔKKGK and Moesin-ΔABD mutant constructs have been described previously (Jiang et al., 2012; Luo et al., 2012).

Cd146-specific siRNA was synthesized by Invitrogen using the following sequences: forward: 5′-GGAGGAGAACCGAGUUCAUTT-3′; reverse: 5′-AUGAACUCGGUUCUCCUCCTT-3′. Scrambled non-targeting control siRNA was used as a negative control. Lipofectamine 2000-mediated transfection was employed

according to the manufacturer's instructions. To downregulate CD146 expression, 50 nM siRNA targeting CD146 was transfected into 10T1/2 cells (pericyte progenitor cells). The co-transfection of CD146-siRNA (50 nM) and a CD146 expression plasmid (*pcDNA3.1-Cd146*, 2 μg) was performed to restore CD146 expression. The empty vector (*pcDNA3.1-empty*) was used as an internal control.

Co-immunoprecipitation

Co-immunoprecipitation was performed as previously described (Jiang et al., 2012). 10T1/2 cells were lysed in a culture dish by adding 500 μl ice-cold RIPA buffer (150 mM NaCl, 50 mM tris, pH 8.0, 0.1% SDS, 0.5% deoxycholate, 1% NP-40, 1 mM phenylmethanesulfonyl fluoride, and a protease inhibitor cocktails) for 40 min. Supernatants were incubated with antibodies at 4 ℃ overnight followed by incubation with protein G-Sepharose (Santa Cruz Biotechnology Inc.) for 4 hours. Immunoprecipitates were washed three times with lysis buffer and analyzed by western blotting using the appropriate antibodies, as indicated.

Cell proliferation and migration assays

Cell proliferation was assayed using the CCK-8 Cell Counting Kit as per manufacturer's instructions. After the appropriate treatments, equal numbers (3,000) of pericytes were seeded into a 96-well plate. Anti-CD146 AA98 or mouse IgG (50 μg/ml) was added to the plate. Forty-eight hours after stimulation with PDGF-B (20 ng/ml), the relative cell number was determined by measuring the optical density of CCK-8 at 450 nm.

Cell migration was assessed using a 96-well Boyden chamber (8-μm pore size;

Corning Costar). After the appropriate treatments, equal numbers (6,000) of pericytes were grown in Dulbecco's modified eagle Medium (with 1% FBS) in the upper chamber. Mouse IgG or anti-CD146 AA98 (50 μg/ml) was added during migration. After stimulation with PDGF-B (20 ng/ml) for 12 hours, cells that had migrated to the lower membrane were stained with crystal violet and counted using a microscope.

Zebrafish husbandry

Adult zebrafish (*Danio rerio*) were maintained according to standard laboratory procedures (Westerfield, 2007). Embryos and larvae were raised in embryo water (5 mM NaCl, 0.17 mM KCl, 0.33 mM $CaCl_2$, 0.33 mM $MgSO_4$), in an incubator at 28.5 ℃. Handling of zebrafish was performed in compliance with "The Legislation of Guangdong Laboratory Animal Management Regulations", and was carefully monitored and approved by the ethics committee of the Affiliated Hospital of Guangdong Medical University. The transgenic lines of tg (*kdrl:egfp*) and tg (*fli1:negfp*) were used for all experiments.

Morpholinos, mRNA synthesis, microinjection and imaging of zebrafish embryos

cd146 MO (AGCAGTGCGGTGTAGGTCATTTCTC), *pdgfrb* MO (ACAGGAACTGAAGTCACTGACCTTC), and control MO (AGGCGTGCGGAGTAGCTCATTTGTC) were purchased from Gene Tools (Philomath, OR, USA) and prepared as 1 μM stock solutions. For *cd146* MO injection, 2 ng of MO was injected at the one-cell stage. Full-length human *CD146* or *CD146-ΔKKGK* cDNA were inserted into the *pCS2+* vector and the mRNA was synthesized *in vitro* using the

mMessagemMachine SP6 Kit (Ambion, USA) according to the instruction manual. For*cd146* rescue experiments, 50 pg*cd146* mRNA and 2 ng *cd146* MO per embryo were co-injected at the one-cell stage. For the dominant negative investigation of CD146-ΔKKGK, 300 pg of mRNA was injected into the yolk at one-cell stage. For the larvae imaging, live embryos were mounted in 1% low-melting agarose and confocal z-stacks images were acquired using Leica TCS SPII 5 confocal microscope (Leica, Solms, Germany).

Whole-mount fluorescent *in situ* hybridization and the quantification of pericytes

For FISH experiment, anti-sense *pdgfrb* probe was synthesized from *pGM-T-pdgfrb* vector with T7 primers. FISH was performed as described (Lauter et al., 2011; Wang et al., 2014). The imaging and quantification of cerebral pericytes were performed by collecting confocal z-stacks of entire larval brains and counting *pdgfrb*$^{+}$ cells from merged images by ImageJ software. Data were evaluated using one-way ANOVA.

BBB permeability assay in zebrafish

For the BBB assay, 70 kDa Rhodamine B-Dextran (2.5 mg/ml in PBS, Molecular Probes, USA) was microinjected into the common cardinal vein of 76-hours post-fertilization tg (*kdrl:egfp*) larvae. Immediately, the larvae were mounted in 1% low-melt agarose (Invitrogen, Carlsbad, CA, USA) and imaged with a Leica TCS SPII 5 confocal microscope (Leica, Solms, Germany).

For BBB permeability assays, Leica software of LAS-AF-Lite was used to calculate the fluorescent intensity value of the vasculature lumen (I) and the fluorescent intensity

value out of the cerebral vasculature (E). The value representing E/I was used for evaluating BBB permeability (Liao et al., 2016). For each analyzed vessel, five positions along the vessel were chosen for calculating this value. The mean of the five E/I values was selected to evaluate the permeability of a single vessel. The anterior (rostral) cerebral vein, middle cerebral vein, posterior (caudal) cerebral vein , primordial midbrain channel, pectoral vein, and basilar artery were chosen for BBB permeability assays in this study (Isogai et al., 2001). Every trial was repeated at least five times.

Statistical analysis

All experiments were performed independently at least three times. The results are shown as the mean ± SEM (standard error of the mean). One or two-way ANOVA tests were used to compare differences between groups in various experiments. Differences with a p-value < 0.05 were considered statistically significant.

References

Isogai, S., Horiguchi, M., and Weinstein, B.M. (2001) The vascular anatomy of the developing zebrafish: an atlas of embryonic and early larval development. Dev Biol 230:278-301

Jiang, T., Zhuang, J., Duan, H., Luo, Y., Zeng, Q., Fan, K., Yan, H., Lu, D., Ye, Z., Hao, J., et al. (2012) CD146 is a coreceptor for VEGFR-2 in tumor angiogenesis. Blood 120:2330-2339

Lauter, G., Soll, I., and Hauptmann, G. (2011) Multicolor fluorescent in situ hybridization to define abutting and overlapping gene expression in the embryonic zebrafish brain. Neural Dev 6:10

Liao, Z., Yang, Z., Piontek, A., Eichner, M., Krause, G., Li, L., Piontek, J., and Zhang, J. (2016) Specific binding of a mutated fragment of Clostridium perfringens enterotoxin to endothelial claudin-5 and its modulation of cerebral vascular permeability. Neuroscience 327:53-63

Luo, Y., Zheng, C., Zhang, J., Lu, D., Zhuang, J., Xing, S., Feng, J., Yang, D., and Yan, X. (2012) Recognition of CD146 as an ERM-binding protein offers novel mechanisms for melanoma cell migration. Oncogene 31:306-321

Wang, Y., Pan, L., Moens, C.B., and Appel, B. (2014) Notch3 establishes brain vascular integrity by regulating pericyte number. Development 141:307-317

Westerfield, M. (2007) The Zebrafish Book. University of Oregon Press

Zhang, Y., Zheng, C., Zhang, J., Yang, D., Feng, J., Lu, D., and Yan, X. (2008) Generation and characterization of a panel of monoclonal antibodies against distinct epitopes of human CD146. Hybridoma (Larchmt) 27:345-352

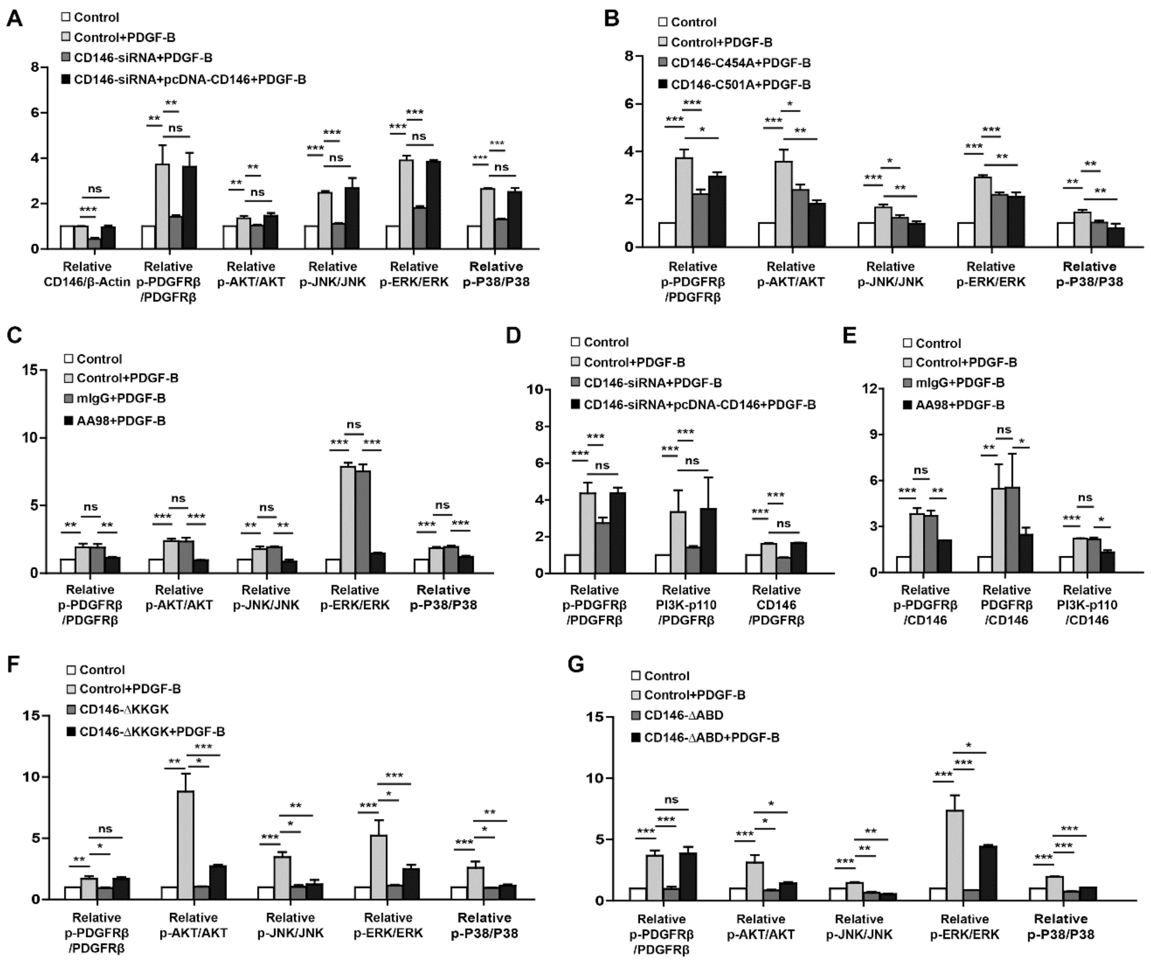

Figure S1. Quantitative analysis of Western blot in Fig. 1. Quantification of the relative p-PDGFRβ/PDGFRβ, p-AKT/AKT, p-JNK/JNK, p-ERK/ERK, and p-p38/p38 index is shown. *$P < 0.05$, **$P < 0.01$, ***$P < 0.001$.

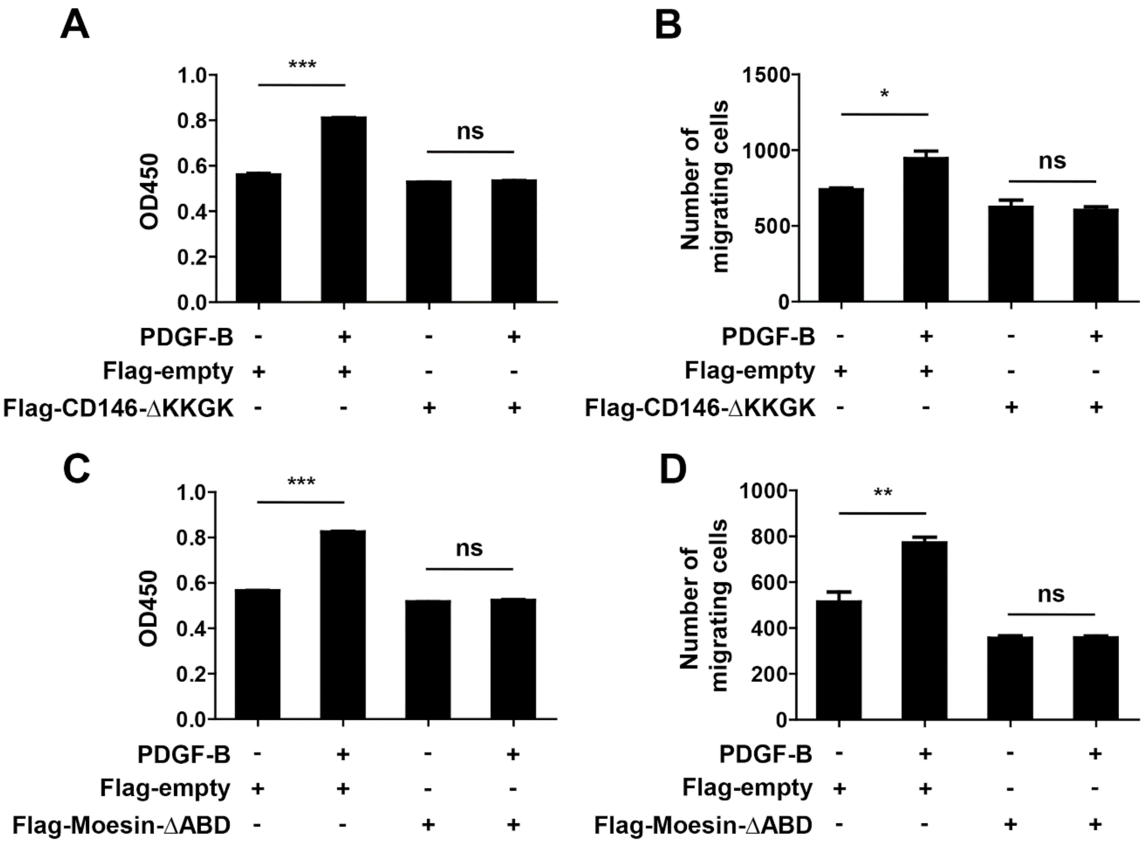

Figure S2. The association of CD146 and cytoskeleton is required for PDGF-B-induced cell proliferation and migration. (A and B) The proliferation and migration of 10T1/2 cells transfected with CD146-ΔKKGK in the presence of PDGF-B (20 ng/ml) were determined by CCK-8 assays (A) and transwell Boyden chamber assays (B), respectively. (C and D) The proliferation and migration of 10T1/2 cells transfected with moesin-ΔABD in the presence of PDGF-B were determined by CCK-8 assays (C) and transwell Boyden chamber assays (D), respectively; **P* < 0.05, ***P* < 0.01, ****P* < 0.001. Data represent three independent experiments.

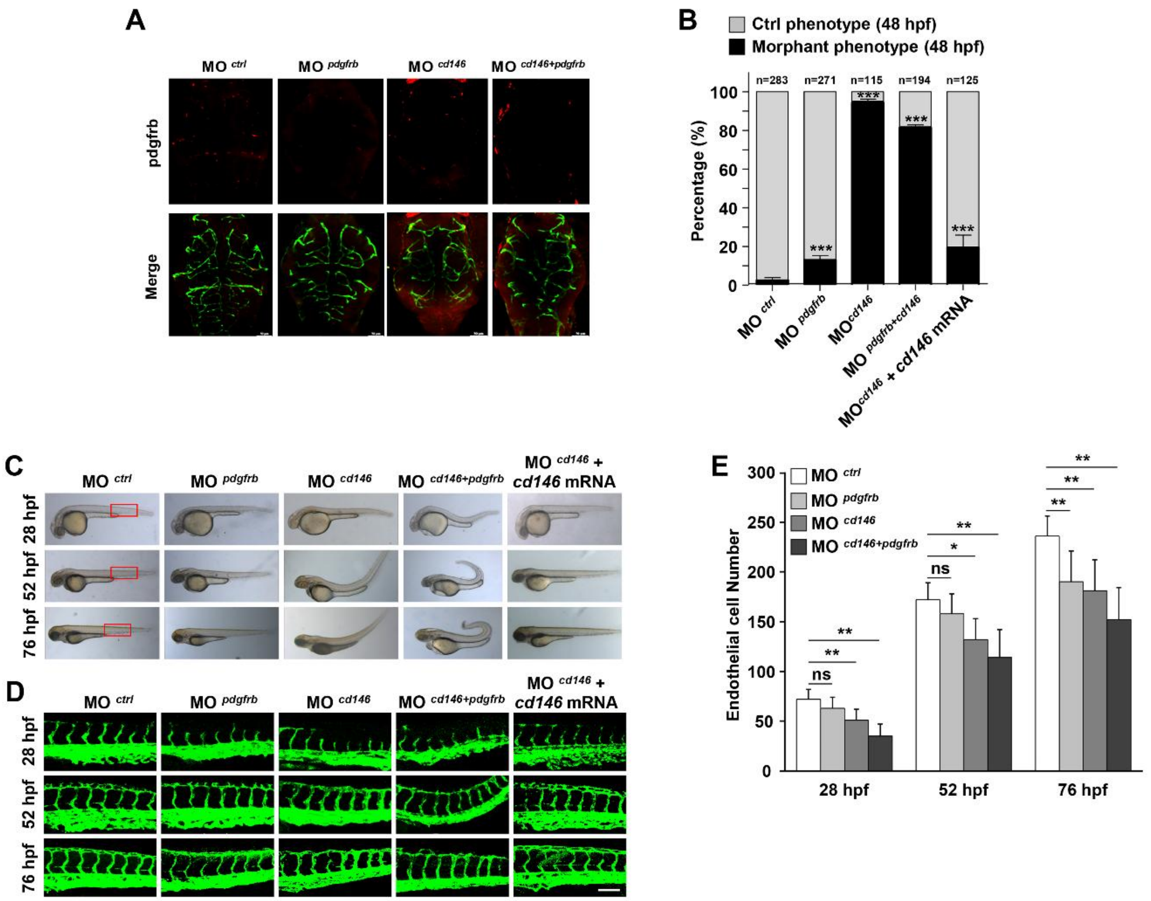

Figure S3. Effects of *cd146* or *pdgfrb* morpholino on zebrafish vascular development. (A) Zebrafish embryos were injected with *cd146* or *pdgfrb* morpholino, co-injected with *cd146* and *pdgfrb* morpholinos, or co-injected with *cd146* morpholino and *cd146* mRNA. *pdgfrb*$^+$ pericytes in zebrafish at 72-hours post-fertilization (hpf) were analyzed to quantify the numbers of recruited pericytes to cerebrovascular walls. (B) Zebrafish embryos were injected with *cd146* or *pdgfrb* morpholino, co-injected with *cd146* and *pdgfrb* morpholinos, or co-injected with *cd146* morpholino and *cd146* mRNA. Quantification of the number of zebrafish with abnormal morphology versus those with normal morphology at 48hpf. (C) Representative morphology of zebrafish injected with morpholinos as indicated. (D) Morpholinos as indicated were injected into transgenic

zebrafish *tg* (*kdrl:egfp*) embryos that expressed endothelial GFP to visualize the vasculature of the trunk region. Vascular patterns (with red box in B) of the zebrafish embryos at 28, 52, and 76 hpf stages were analyzed. Scale bar represents 100 μm. (E) Morpholinos as indicated were injected into transgenic zebrafish *tg (fli1:negfp)* embryos that expressed nuclear GFP to enable visualization of endothelial cells (ECs). The number of ECs in zebrafish at 28, 52, and 76 hpf stages were analyzed; $*P < 0.05$, $**P < 0.01$, $***P < 0.001$. Data represent three independent experiments.
